# Not Only Follicular Helper T‐Cells but Also Peripheral Helper T‐Cells Expanded Correlate With Disease Severity and B‐Cell Differentiation in Graves’ Disease

**DOI:** 10.1155/ije/8591694

**Published:** 2026-06-16

**Authors:** Zhaowei Huang, Yanfei Jiang, Xinwei Zhang, Xiaorong Yang, Ronghua Song, Jin-an Zhang

**Affiliations:** ^1^ Graduate School, Shanghai University of Traditional Chinese Medicine, Shanghai, 201203, China, shutcm.edu.cn; ^2^ Department of Endocrinology & Rheumatology, Shanghai University of Medicine & Health Sciences Affiliated Zhoupu Hospital, Shanghai, 201318, China, sumhs.edu.cn

**Keywords:** B-cell, Graves’ disease, lymphoid follicle, Tfh cell, thyroid tissue, Tph cell

## Abstract

**Objective:**

To study the expression of peripheral helper T (Tph)‐cells in the blood and thyroid of Graves’ disease (GD) patients and their correlation with B‐cells and clinical indicators.

**Methods:**

A total of 25 cases of newly diagnosed GD and 25 cases of healthy controls were recruited. Peripheral blood mononuclear cells were isolated. The frequency and proliferative activity of follicular helper T (Tfh)‐cells, Tph cells, and B‐cells were detected by flow cytometry. Thyroid tissues of 4 patients with GD and 4 normal thyroid tissues were selected for multiple immunofluorescence staining. The number of Tfh cells and Tph cells in the thyroid tissue was observed.

**Results:**

Tph and Tfh cells were significantly increased in the blood and thyroid tissue of GD patients compared with healthy controls. Both cells were positively correlated with the proliferative activity of B‐cells. The proportion of Tph cells correlated with serum TT4 (*r* = 0.501) and TR‐Ab (*r* = 0.472) levels. Tph cell levels were significantly increased in the TR‐Ab (high) group compared to the TR‐Ab (low) group. Tph is localized in areas beyond lymphoid follicles and may extensively promote B‐cells in the inflamed thyroid gland.

**Conclusion:**

This study demonstrates for the first time that Tph cells are significantly expanded in the blood and thyroid of GD patients. Similar to Tfh cells, Tph cells correlated with GD severity and B‐cell differentiation. Therefore, it may be a new target for GD immunotherapy.

## 1. Introduction

Graves’ disease (GD), also known as toxic diffuse goiter, is primarily manifested by hyperthyroidism. It is an autoantibody‐mediated autoimmune disease. The pathological feature of GD is that lymphocytes infiltrate thyroid tissue and produce thyrotropin receptor antibodies (TR‐Ab) and inflammatory cytokines such as interferon‐γ (IFN‐γ) and tumor necrosis factor‐α (TNF‐α). GD is commonly seen in females between the ages of 30 and 50 years, with a male‐to‐female incidence ratio of approximately 1:4 [1]. The pathogenesis of the disease is not fully understood, and the proliferation and activation of B‐cells play a key role in it. Therefore, elucidating the differentiation mechanism of B‐cells and thus inhibiting their autoantibody secretion is important for the development of immunotherapy strategies for GD.

Peripheral helper T (Tph) cells are a recently established subset of CD4+ B helper T‐cells. They not only share a similar transcriptome to follicular helper T (Tfh)‐cells but also have unique characteristics and functions. Tph cells and Tfh cells share many functions related to B‐cell assistance, including recruitment of B‐cells to ectopic lymphoid follicles and induction of B‐cell differentiation into antibody‐producing cells [2]. The difference is that Tfh cells interact with B‐cells within lymphoid follicles, whereas Tph cells are localized in the periphery. Tph cells do not express C‐X‐C chemokine receptor Type 5 (CXCR5) and are more widely distributed in inflamed tissues to assist B‐cells [3]. Atypical memory B‐cells (CD11c+CD21‐CXCR5‐) that also lack CXCR5 expression may be one of the target B‐cells for Tph cells [4, 5]. Tph cell populations (PD‐1hi CXCR5‐ CD4+) were found to be significantly increased in the synovium of rheumatoid arthritis (RA) patients. They are able to express factors that aid B‐cell differentiation such as C‐X‐C motif chemokine Ligand 13 (CXCL13), Interleukin‐21 (IL‐21), inducible costimulatory molecules (ICOS), and proto‐oncogene c‐Maf (C‐MAF) [6]. Tph cells are uniquely suited to promote B‐cell responses and antibody production in the nonlymphoid tissues of inflamed organs. In addition to this, Tph cell expansion has been found in many other autoimmune diseases and correlates with disease severity. These include systemic lupus erythematosus (SLE), IgG4‐related diseases, IgA nephropathy, Type 1 diabetes (T1D), and Sjogren’s syndrome [7–12]. Although Tph cells are beginning to gain prominence as a new cell subpopulation, their role in GD is unclear. Therefore, we investigated the expression of Tph cells in the blood and thyroid of GD patients and their correlation with B‐cells and clinical indicators.

## 2. Materials and Methods

### 2.1. Participants

We recruited 25 new‐onset GD patients and 25 age‐ and sex‐matched healthy controls (HC) for flow cytometry experiments from February 2024 to June 2024. The thyroid tissues of four surgically resected GD patients were stained by Multiplex immunofluorescence (mlF). Four normal thyroid tissues were obtained from paratumoral tissues from patients with benign thyroid adenoma. All patients were from the Endocrinology department or thyroid gland Surgery Department of Shanghai Zhoupu Hospital. Peripheral blood samples of the GD group were obtained from newly diagnosed patients who had not taken any antithyroid drugs (including methimazole or propylthiouracil). The four surgically resected tissue samples were from GD patients with a poor response to long‐term antithyroid drug therapy. All HCs received blood samples from the same hospital’s medical examination center The study was approved by the Ethics Committee of Zhoupu Hospital (2023‐NSFC‐17–610302196504170039), and all participants signed an informed consent form.

The diagnosis of GD should be made by combining clinical manifestations and laboratory findings [13]. Clinical symptoms and signs include irritability, agitation, heat intolerance, excessive sweating, weight loss, muscle weakness, hyperphagia, and diffuse goiter. Laboratory tests include serum total thyroxine (TT4), free thyroxine (FT4) serum total triiodothyronine (TT3), free triiodothyronine (FT3), thyroid‐stimulating hormone (TSH), TR‐Ab and thyroid peroxidase antibodies (TPO‐Ab). All laboratory tests were performed in the central laboratory of Zhoupu Hospital. Participant exclusion criteria included age < 18 years, history of antithyroid medication, history of thyroid hormone analog medication, serious infections, serious cardiorespiratory disease (such as coronary artery disease and chronic obstructive pulmonary disease), and breastfeeding or pregnancy.

### 2.2. Flow Cytometry

Approximately 10 mL of venous blood was collected from each participant using heparin anticoagulant tubes. Peripheral blood mononuclear cells (PBMCs) were isolated by Ficoll density gradient centrifugation according to the instructions of lymphocyte separation solution (DAKEWE, Beijing, China). The isolated PBMCs were stored at −80°C for flow cytometry experiments. The blank group, single‐positive control group, Fluorescence Minus One (FMO) group, and an experimental group were set up. Each set of samples were incubated with 1 μL of live‐dead dye for 15 min at room temperature and protected from light, then centrifuged at 500 g and washed. Then, appropriate amount of cell surface fluorescent antibody was added and incubated at room temperature for 15 min away from light. Antibodies include CD3‐APC‐Cy7, CD4‐FITC, CXCR5‐BV421, PD‐1‐BV605, Ki67‐APC, CD25‐PE‐Cy7, and CD19‐PERCP‐CY5.5 (Supporting Table 1. Antibodies used in flow cytometry). Finally, cells were washed with precooled wash buffer. Detection was performed by flow cytometry (BD Fortessa X20) within 3 h. Data from flow cytometry experiments were analyzed using FlowJo software (Version 10.6).

### 2.3. mlF

mlF is an enzymatic assay for in situ labeling of target proteins. The method allows for the simultaneous detection of multiple target proteins on a single tissue sample and is widely used to identify and localize different cell types within tissue samples. The paraffin sections were hydrated with xylene and different concentrations of alcohol. Paraffin sections are hydrated with xylene and various concentrations of alcohol, then follow these steps to complete the mIF. (1) Antigen repair: pH 9.0, preheat in microwave at high temperature for 5 min, place the sample and repair at low temperature for 20 min (2) Containment: 3% hydrogen peroxide for 10 min, 5% bovine serum albumin (BSA) for 10 min (3) Incubation: dilute the antibody according to the instructions, incubate the primary antibody for 60 min, the secondary antibody for 10 min, and the fluorescent dye TSA for 10 min. Repeat Steps 1–3 three times, Round 1: PD1 antibody, YX TSA‐520; Round 2: CXCR5 antibody, YX TSA‐670; Round 3: CD4 antibody, YX TSA‐570 (Supporting Table 2. Reagents used in mlF). The final DAPI was incubated for 10 min at room temperature and antifluorescence quencher was added. The slices were sealed and photographed using a fluorescence microscope.

### 2.4. Statistical Analysis

Statistical analyses were performed using *SPSS25.0*. Continuous variables with a normal distribution and homogeneity of variance were represented by ($\overset{¯}{X}$ *± S*), and comparisons between groups were performed by *t*‐test. Continuous variables that do not conform to the normal distribution are represented by the median (interquartile range) (M [P25‐P75]) and comparisons between groups were made by the Mann–Whitney *U* test. Categorical variables are expressed in terms of quantity and percentage (*n*, %), and the *χ* ^2^ test was used. In the statistical tests of the above methods, *p* < 0.05 was considered significant.

## 3. Results

### 3.1. Increased Tfh and Tph Cells in Peripheral Blood

**FIGURE 1:**
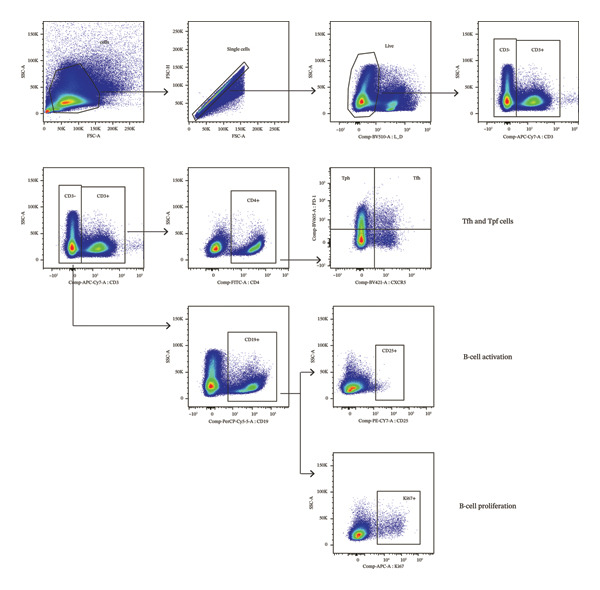
Gating strategy for flow cytometry. Tfh cells (CD3+CD4+PD‐1+CXCR5+), Tph cells (CD3+CD4+PD‐1+CXCR5‐), B‐cells (CD3‐CD19+), activation marker (CD25+), and proliferation marker (Ki67+).

**FIGURE 2:**
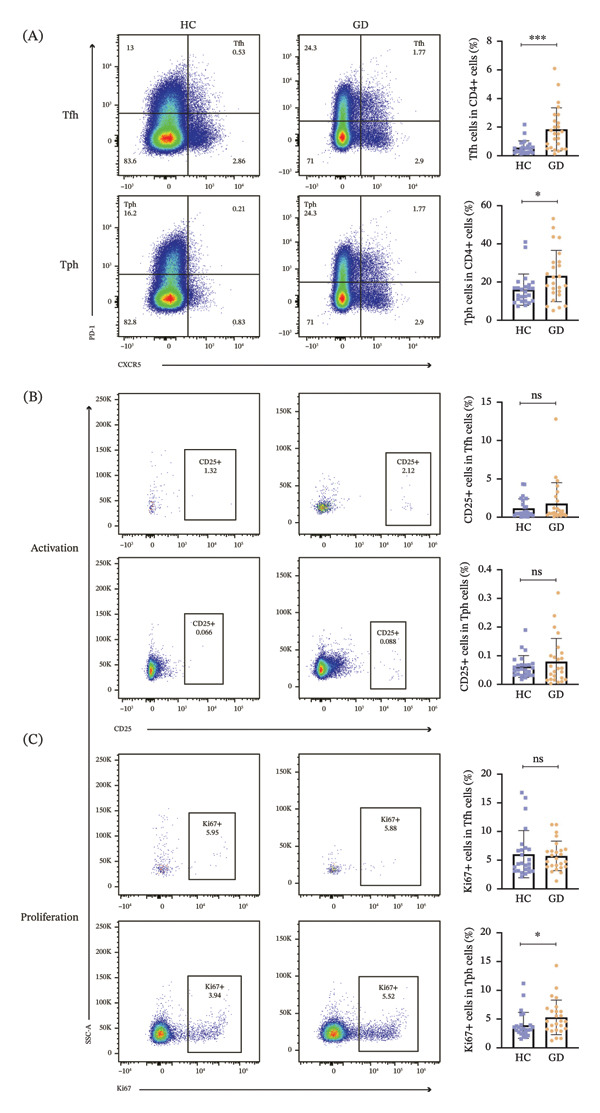
Differences in Tfh and Tph cells between the two groups. (A) Frequency of Tfh and Tph cells. (B) Degree of Tfh and Tph cell activation. (C) Proliferative activity of Tfh and Tph cells. (ns: *p* ≥ 0.05, ^∗^: *p* < 0.05, ^∗∗∗^: *p* < 0.001, Tfh: follicular helper T, Tph: peripheral helper T, HC: healthy controls, GD: Graves’ disease).

A total of 50 participants were recruited for the study (Table 1). The frequency, activation level, and proliferative activity of different lymphocyte subtypes were detected by multicolor flow cytometry (Figure 1). The results showed that the proportions of both Tfh (CD4+PD‐1+CXCR5+) and Tph (CD4+PD‐1+CXCR5‐) cells were significantly increased in the GD group compared to the HC group (*p* < 0.05) (Figure 2A). In particular, Tph cells with proliferative activity (Ki67+) were significantly higher in the GD group than in the HC group (*p* < 0.05) (Figure 2C). There was no significant difference in the activation levels (CD25+) of Tfh and Tph cells between the two groups (Figure 2B).

**TABLE 1:** Clinical characteristics of study participants.

Variables	Reference interval	GD (*n* = 25)	HC (*n* = 25)	*p* value
Age (years)	/	37.04 ± 12.70	36.64 ± 12.02	0.909
Female, *n* (%)	/	18 (72)	16 (64)	0.544
TT3 (nmol·L^−1^)	1.21–3.01	7.00 (4.47–9.43)	2.30 (1.82–2.70)	< 0.001
TT4 (nmol·L^−1^)	71.31–165.06	239.86 ± 58.95	124.28 ± 29.85	< 0.001
FT3 (pmol·L^−1^)	3.15–6.70	31.86 ± 12.48	4.94 ± 1.04	< 0.001
FT4 (pmol·L^−1^)	11.92–21.62	71.90 (50.20–100.00)	17.35 (14.37–19.42)	< 0.001
TSH (uIU·mL^−1^)	0.300–5.000	0.005 (0.005–0.005)	1.827 (0.789–3.207)	< 0.001
TG (ng·mL^−1^)	1.59–50.03	11.08 (1.00–122.90)	2.85 (1.50–4.26)	0.185
TG‐Ab (U·mL^−1^)	< 115.00	95.30 (23.95–385.50)	19.35 (11.31–32.44)	< 0.001
TR‐Ab (IU·L^−1^)	< 1.70	11.90 (3.77–32.20)	0.44 (0.31–0.65)	< 0.001
TPO‐Ab (U·mL^−1^)	< 34	162.0 (18.5–470.0)	11.35 (6.8–19.2)	< 0.001

*Note:* TT3: total triiodothyronine, TT4: total thyroxine, FT3: free triiodothyronine, FT4: free thyroxine, TG: thyroglobulin, TG‐Ab: thyroglobulin antibodies, TR‐Ab: thyroid‐stimulating hormone receptor antibodies, and TPO‐Ab: thyroid peroxidase antibodies.

Abbreviations: GD, Graves’ disease; HC, healthy control; TSH, thyroid‐stimulating hormone.

### 3.2. Increased Proliferative Activity of B‐Cells in Peripheral Blood

**FIGURE 3:**
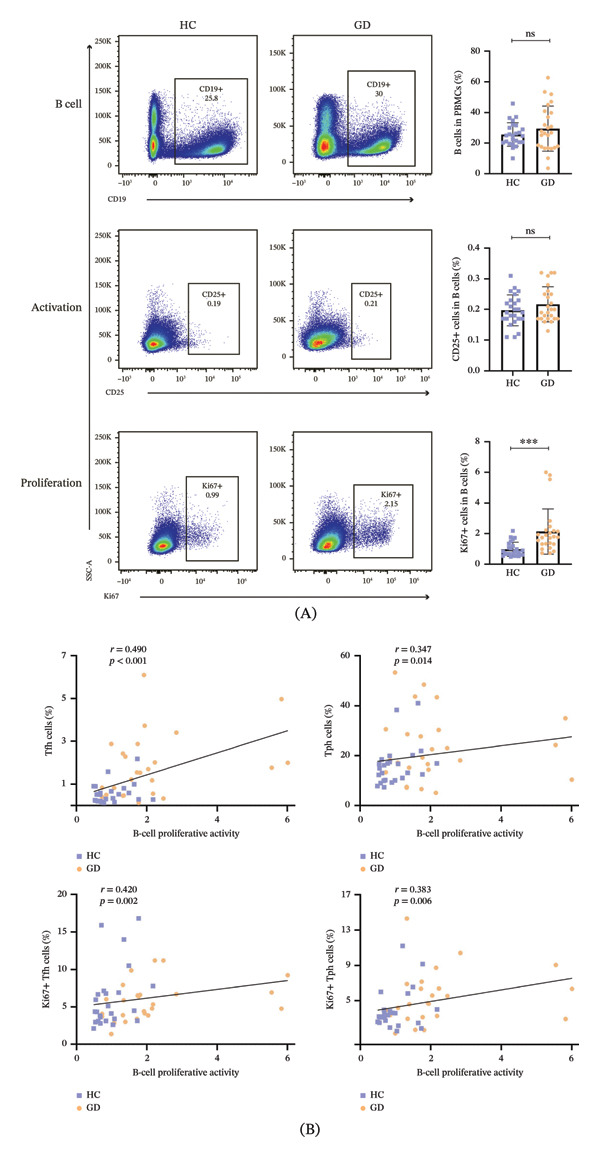
B‐cell correlation with Tfh and Tph cells. (A) Differences in B‐cells between the two groups. (B) B‐cell proliferative activity correlates with Tfh and Tph cells. (ns: *p* ≥ 0.05, ^∗∗∗^: *p* < 0.001, Tfh: follicular helper T, Tph: peripheral helper T, HC: healthy controls, and GD: Graves’ disease).

There was no significant difference in the proportion of B‐cells (CD3‐CD19+) in the GD group compared with the HC group. However, in the GD group, the B‐cells with proliferation potential increased significantly (*p* < 0.05) (Figure 3A). Proliferating B‐cells were positively correlated with Tfh (*r* = 0.490, *p* < 0.001) and Tph (*r* = 0.347, *p* = 0.014) cells. Also, the proliferation degree of B‐cells was positively correlated with the proliferation level of Tfh (*r* = 0.420, *p* = 0.002) and Tph (*r* = 0.383, *p* = 0.006) cells (Figure 3B).

### 3.3. Tph Cells Correlate With Disease Severity

**FIGURE 4:**
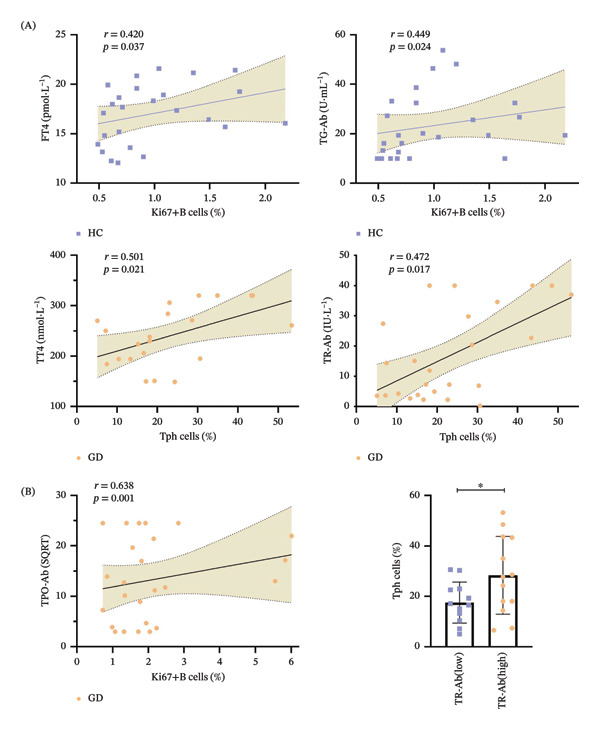
The correlation between lymphocyte subpopulations and clinical indicators. (A) Correlation of lymphocyte subpopulations with FT4, TG‐Ab, TT4, TR‐Ab, and TPO‐Ab. (B) Differences in Tph cells between TR‐Ab (high) and TR‐Ab (low) groups (grouped by the median TR‐Ab). (Tph: peripheral helper T, HC: healthy control; GD: Graves’ disease, FT4: free thyroxine, TG‐Ab: thyroglobulin antibodies, TT4: total thyroxine; TR‐Ab: thyroid‐stimulating hormone receptor antibodies; TPO‐Ab: thyroid peroxidase antibodies, ^∗^: *p* < 0.05).

Correlation analysis between lymphocyte subpopulations and clinical indicators showed (Table 2) that FT4 and TG‐Ab levels in the HC group were significantly correlated with the proliferative activity of B‐cells (FT4: *r* = 0.420, *p* = 0.037; TG‐Ab: *r* = 0.449, *p* = 0.024) (Figure 4A). TT4 levels were significantly correlated with the proportion of Tph cells in GD patients (*r* = 0.501, *p* = 0.021). TR‐Ab levels were also positively correlated with Tph cells (*r* = 0.472, *p* = 0.017). TPO‐Ab levels were correlated with the proliferative activity of B‐cells (*r* = 0.638, *p* = 0.001) (Figure 4A). Stratified by the median level of TR‐Ab (high: TR‐Ab ≥ 11.9 IU L^−1^; low: TR‐Ab < 11.9 IU L^−1^), Tph cell levels were significantly increased in the TR‐Ab (high) group compared to the TR‐Ab (low) group (*p* < 0.05) (Figure 4B).

**TABLE 2:** The correlation between lymphocyte subpopulations and clinical indicators.

**Correlation spearman (r)**	**HC group**					
	**Tfh**	**Ki67+ Tfh**	**Tph**	**Ki67+ Tph**	**B-cells**	**Ki67+ B-cells**

TT3	0.226	−0.124	0.094	−0.065	−0.132	0.237
TT4	0.129	−0.137	0.150	−0.071	−0.058	0.235
FT3	0.048	−0.140	0.089	−0.138	−0.132	0.274
FT4	0.174	−0.073	0.215	−0.113	−0.177	0.420^∗^
TSH	−0.150	0.151	−0.157	0.061	0.162	−0.28
TG	−0.106	0.055	0.011	0.166	−0.134	0.353
TG‐Ab	0.104	0.034	0.034	0.153	−0.236	0.449^∗^
TR‐Ab	0.145	−0.005	0.168	0.036	−0.009	0.359
TPO‐Ab	0.152	−0.011	0.156	0.042	−0.016	0.361

**Spearman’s correlation (r)**	**GD group**					
	**Tfh**	**Ki67+ Tfh**	**Tph**	**Ki67+ Tph**	**B cells**	**Ki67+ B-cells**

TT3	0.046	0.380	0.373	−0.278	−0.145	0.275
TT4	0.052	0.287	0.501^∗^	−0.274	−0.225	0.266
FT3	0.024	0.371	0.278	−0.153	−0.218	0.172
FT4	−0.040	0.273	0.244	−0.163	−0.286	0.14
TSH	0.311	−0.057	0.226	−0.255	0.113	0.311
TG	0.167	−0.020	−0.113	0.148	0.178	0.016
TG‐Ab	−0.168	0.068	0.125	−0.045	0.267	0.158
TR‐Ab	0.269	0.228	0.472^∗^	−0.200	−0.108	0.226
TPO‐Ab	0.069	0.308	0.162	0.044	−0.171	0.638^∗∗∗^

*Note:* Tfh: follicular helper T, Tph: peripheral helper T, TT3: total triiodothyronine, TT4: total thyroxine, FT3: free triiodothyronine, FT4: free thyroxine, TSH: thyroid‐stimulating hormone, TG: thyroglobulin, TG‐Ab: thyroglobulin antibodies, TR‐Ab: thyroid‐stimulating hormone receptor antibodies, TPO‐Ab: thyroid peroxidase antibodies.

Abbreviations: GD, Graves’ disease; HC, healthy control.

^∗^ *p* < 0.05.

^∗∗∗^ *p* < 0.001.

### 3.4. Increased Tfh and Tph Cells in the Thyroid

**FIGURE 5:**
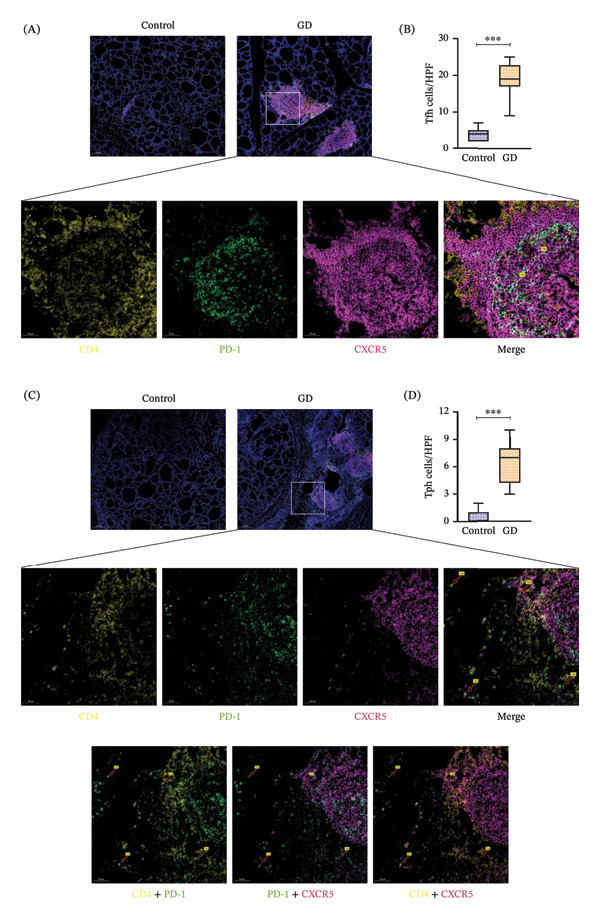
Multiplex immunofluorescence of Tfh and Tph cells in thyroid tissue. (A) Representative pictures of Tfh cells (white arrows). (B) Tfh cell counts (*n* = 12, 3 HPF per sample). (C) Representative pictures of Tph cells (red arrows). (D) Tph cell counts (*n* = 12, 3 HPF per sample). (CD4: yellow, PD‐1: green, CXCR5: pink, nucleus: blue, ^∗∗∗^: *p* < 0.001, GD: Graves’ disease, and HPF: high power field).

The mlF results showed that a large number of lymphocytes were infiltrated and clustered in the thyroid of GD patients. Tfh and Tph cells were found in all four GD thyroid samples. None or only occasional lymphocytic infiltration was seen in the thyroid gland of the control group. Both Tfh and Tph cells were significantly increased in the thyroid glands of GD patients compared with the control group (*n* = 12, 3 high power field per sample, *p* < 0.001) (Figure 5B,D). A large number of Tfh cells were concentrated inside the lymphoid follicles (Figure 5A). However, Tph cells were more widely distributed, and Tph cells were found at the edge of the lymphoid follicles and outside the lymphoid follicles (Figure 5C).

## 4. Discussion

Hyperthyroidism is caused by an increase in self‐synthesis and secretion of thyroid hormones. The overall prevalence of hyperthyroidism is about 1.3%, of which more than 70% is due to GD [14]. At present, antithyroid drugs, radioactive iodine, and surgical resection are the main treatment methods for GD. There are also a number of treatment‐related side effects and complications, including granulocyte deficiency, radiation thyroiditis, and recurrent laryngeal nerve injury [14]. Therefore, it is urgent and attractive to elucidate the pathogenesis of GD. The thyroid gland of GD patients is infiltrated with a large number of T lymphocytes. On the one hand, these T lymphocytes produce inflammatory cytokines such as IFN‐γ, TNF‐α, and IL‐17A [15]. On the other hand, they help B‐cells in lymphoid follicles to proliferate and differentiate into cells that secrete autoantibodies. This is necessary for chronic immune inflammation. Identifying the mechanisms and drivers behind B‐cell proliferation is a key focus in GD research. Previous studies have shown that Tfh cells are involved in the process of B‐cell differentiation, assisting in the activation of B‐cells and promoting the formation of germinal centers [15, 16]. In the present study, we not only once again verified the increased expression and infiltration of Tfh cells in GD. Importantly, we found for the first time that Tph cells expanded as significantly as Tfh cells in peripheral blood and thyroid of GD patients. Both of them are associated with disease severity and B‐cell proliferative activity. These results suggest that Tph cells may play a key role in the pathogenesis of GD.

In the immune system, CD4+ T‐cells help B‐cells and antibody immune responses. In particular, Tfh cells are important for the establishment and refinement of humoral immunity and play an important role in autoimmune and tumor‐related diseases. CD4+ Tfh cells express CXCR5, PD‐1, ICOS, the cytokine IL‐21, and the transcription factors C‐MAF and BATF, while B‐cells express CXCL3, a ligand of CXCR5 [17]. CXCR5+ causes Tfh cells to homing in B‐cell follicles. Through B‐T cell interactions and secretion of associated cytokines promote germinal center formation and drive the development of high‐affinity antibodies [17]. Tfh cells are associated with autoantibody‐mediated diseases. Patients with SLE have an increased frequency of activated Tfh cells in the blood [18]. Ectopic germinal centers and Tfh cells are frequently found in the kidneys of patients with lupus nephritis [19]. He et al. found that low‐dose IL‐2 is an effective treatment for SLE because IL‐2 is a potent inhibitor of Tfh cells [20]. We found that Tfh cells expanded in GD blood and diseased thyroid, and the degree of expansion was correlated with the severity of the disease. Our findings were similar to those of Tfh cells in other autoimmune diseases. Tfh cells are also strongly associated with disease activity in RA, Sjogren’s syndrome, T1D, and myasthenia gravis [21]. Tfh cells (PD‐1+ CXCR5+) correlate with disease activity score (DAS28) and anti‐CCP antibody levels in RA [22].

A recent series of autoimmune disease studies have established a new subpopulation of CD4+ B helper T‐cells called peripheral helper T‐cells in human tissue and blood samples. Similar to Tfh cells, Tph cells (PD‐1hi CXCR5‐CD4+) also assist B‐cell function via CD84 and IL‐21. However, because Tph cells do not express CXCR5 but express chemokine receptors CCR2, CCR5, and CX3CR1, Tph cells locate inflammatory tissues rich in CCL2, CCL5, and CX3CL1. That is, it is located at the periphery of the tertiary lymphatic structure rather than in the center, thus providing more extensive help to B‐cells in inflammatory tissue. Rao et al. found that in the joints of RA patients, Tph cells can help memory B‐cells as effectively as Tfh cells [6]. An increase in Tph cells was also found in SLE and was much higher in the disease active group than in the inactive group [7]. Another similar study concluded that the increase in Tph cells in SLE patients not only correlates with disease activity but also helps B‐cell differentiation and promotes autoantibody production [8]. Increased Tph cells have been found in the peripheral blood of patients with T1D and IgA nephropathy and correlate with disease severity [10, 11]. Tph cells are enriched in both peripheral blood and salivary glands of Sjogren’s syndrome patients and promote the formation of ectopic lymphoid structures [12]. Excitingly, our study is the first to detect Tph cell expansion in GD patients. It was also positively correlated with the proliferative activity of B‐cells and TR‐Ab levels.

Notably, the frequencies of Tph and Tfh cells are positively correlated in RA, SLE, and HC [6, 23, 24]. This suggests that Tph and Tfh cells codevelop in our body in both healthy and diseased states. Characteristics associated with B‐cell helper function are to a large extent the same for both. Both subpopulations express PD‐1, IL‐21, and CXCL13. Autoantibodies are produced by plasma cells, but at the same time memory B‐cells are the second line of defense for antibody immunity. Upon encountering, the same antigen again, memory B‐cells are able to differentiate more rapidly into antibody‐secreting effector cells [25]. Long‐lived plasma cells and high‐affinity memory B‐cells are usually produced in secondary lymphoid organs and are dependent on the help of Tfh cells [25]. However Tph cells colocalize with B‐cells outside lymphoid follicles in inflamed tissues [6]. Atypical memory B‐cells (CD11c + CD21‐CXCR5‐), which similarly lack CXCR5 expression, reside in extrafollicular regions [2, 26] and may represent one of the key target B‐cell subsets for Tph cells. Consistent with this, our study yielded parallel findings: in the thyroid tissues of GD patients, Tfh cells were predominantly clustered within the lymphoid follicles, whereas Tph cells exhibited a much broader distribution pattern and were readily detected in extrafollicular areas. This finding indicates that, unlike Tfh cells, Tph cells may mediate B‐cell helper activity across a wider spectrum of inflamed tissue sites. One study found that both CD11c + CD21‐CXCR5‐B‐cells and Tph cells were present in lupus nephritis tissues [5, 27]. And the frequency of CD11c + CD21‐CXCR5‐B‐cells in SLE blood was highly correlated with Tph cells [5, 24].

This study has several strengths. First, to our knowledge, this is the first study to systematically investigate the expansion, spatial distribution characteristics, and clinical correlation of Tph cells in the peripheral blood and thyroid target organs of patients with GD, which fills the research gap regarding the role of Tph cells in the pathogenesis of GD. Second, this study combined multiparameter flow cytometry for the detection of circulating immune cells and multiplex immunofluorescence staining for in situ tissue localization. We not only confirmed the abnormal expansion of Tph cells in the systemic circulation but also clarified their wide distribution in the extrafollicular regions of inflamed thyroid tissue. Compared with studies focusing only on peripheral blood, our work provides more sufficient pathological evidence for the proinflammatory effect of Tph cells in GD. Finally, this study identified the positive correlation between Tph cells and B‐cell proliferative activity, as well as key clinical indicators of GD severity, providing a novel potential target for GD immunotherapy and laying a theoretical foundation for subsequent mechanistic research.

Limitations of this study should also be acknowledged. First, this is a single‐center study with a relatively small sample size, which may limit the statistical power of the study. Therefore, our findings need to be further verified in multicenter cohorts with larger sample sizes. Second, this study is an observational clinical study. Further in vitro and in vivo functional experiments are required to verify the specific molecular mechanism by which Tph cells regulate B‐cell differentiation and promote the progression of GD. Meanwhile, colocalization staining should be performed to identify the specific target B‐cell of Tph cells in thyroid tissue (such as CXCR5‐atypical memory B‐cells), which will be supplemented and improved in our subsequent studies.

## 5. Conclusion

In conclusion, our study found for the first time a significant increase in Tph cells in peripheral blood and thyroid in GD. Similar to Tfh cells, Tph cells were associated with GD severity and B‐cell differentiation. Therefore, Tph cells may be a new perspective to study the pathogenesis of GD and a new target for immunotherapy.

## Author Contributions

Zhaowei Huang: conceptualization, investigation, methodology, visualization, and writing–original draft. Yanfei Jiang: data curation, formal analysis, investigation, and writing–original draft. Xinwei Zhang: methodology, software, and visualization. Xiaorong Yang: methodology, supervision, and validation. Ronghua Song: funding acquisition, project administration, and writing–review and editing. Jin‐an Zhang: funding acquisition, resources, and writing–review and editing.

## Funding

This research was funded by the National Natural Science Foundation of China (82370791) and the Scientific Research Program of Shanghai Pudong New Area Health Commission (the General Program, PW2023A‐22).

## Ethics Statement

The study was approved by the Ethics Committee of Shanghai University of Medicine & Health Sciences Affiliated Zhoupu Hospital (2023‐NSFC‐17–610302196504170039), and all participants signed an informed consent form. All research procedures involving human participants were in accordance with the 1964 Declaration of Helsinki and its subsequent revisions.

## Conflicts of Interest

The authors declare no conflicts of interest.

## Supporting Information

Additional supporting information can be found online in the Supporting Information section.

## Supporting information


**Supporting Information 1** The provider, product number, and detailed dosage of the reagents used in the research are listed in the supporting table. Supporting Table 1: Antibodies used in flow cytometry.

**Supporting Information 2** Supporting Table 2: Reagents used in multiplex immunofluorescence.

## Data Availability

Raw data supporting the results of this study were uploaded to the figshare data sharing platform. They can be downloaded via the following link (https://doi.org/10.6084/m9.figshare.28692374).
