# Supplementary material for: Not Only Follicular Helper T‐Cells but Also Peripheral Helper T‐Cells Expanded Correlate With Disease Severity and B‐Cell Differentiation in Graves’ Disease
Source: Int J Endocrinol. 2026 Jun 16;2026:8591694. doi: 10.1155/ije/8591694 (PMC13270354; doi:10.1155/ije/8591694)
Supplement: Supplementary file 1 — Supporting Information 1 The provider, product number, and detailed dosage of the reagents used in the research are listed in the supporting table. Supporting Table 1: Antibodies used in flow cytometry. [file IJE-2026-8591694-s001.doc]

Supplementary Table 1. Antibodies used in flow cytometry.

| Antibodies | Provider | Reference | Dosages |
| --- | --- | --- | --- |
| Live-Dead-BV510 | BD | 564406 | 1 µl/test |
| CD3-APC-Cy7 | BD | 557832 | 2.5 µl/test |
| CD4-FITC | BD | 555346 | 10 µl/test |
| CXCR5-BV421 | BD | 562747 | 2.5 µl/test |
| PD-1-BV605 | BD | 563245 | 2.5 µl/test |
| Ki67-APC | BD | 558615 | 2.5 µl/test |
| CD25-PE-Cy7 | BD | 557741 | 2.5 µl/test |
| CD19-PERCP-CY5.5 | BD | 566396 | 2.5 µl/test |
