# Supplementary material for: Not Only Follicular Helper T‐Cells but Also Peripheral Helper T‐Cells Expanded Correlate With Disease Severity and B‐Cell Differentiation in Graves’ Disease
Source: Int J Endocrinol. 2026 Jun 16;2026:8591694. doi: 10.1155/ije/8591694 (PMC13270354; doi:10.1155/ije/8591694)
Supplement: Supplementary file 2 — Supporting Information 2 Supporting Table 2: Reagents used in multiplex immunofluorescence. [file IJE-2026-8591694-s002.doc]

Supplementary Table 2. Reagents used in multiplex immunofluorescence.

| Reagents | Provider | Reference | Concentration |
| --- | --- | --- | --- |
| CXCR5 antibody | Abcam | ab254415 | 1:7500 |
| CD4 antibody | Abcam | ab133616 | 1:400 |
| PD1 antibody | sino biological | 10377-MM23 | 1:400 |
| YX TSA 570 | WiSee | D110013 | / |
| YX TSA 520 | WiSee | D110011 | / |
| YX TSA 670 | WiSee | D110017 | / |
